# Antimicrobial and Antioxidant Activities of Different Extracts from Different Parts of *Zilla spinosa* (L.) Prantl

**DOI:** 10.1155/2020/6690433

**Published:** 2020-12-03

**Authors:** Mohamed H. A. Suleiman, Ali A. Ateeg

**Affiliations:** ^1^Department of Chemistry, College of Science, King Khalid University, Abha 61413, Asir, Saudi Arabia; ^2^Department of Chemistry, Faculty of Science, University of Kordofan, El Obeid, Sudan; ^3^Department of Chemistry, College of Education, El Imam El Mahdi University, Kosti, Sudan

## Abstract

*Zilla spinosa* is commonly used in traditional medicine to treat gastrointestinal disorders and diabetes. In this study, aqueous ethanol (AE) and aqueous methanol (AM) extracts from aerial parts and roots of *Z. spinosa* were investigated. The total phenolic (TPC) and flavonoid (TFC) contents and antioxidant capacities in terms of 1,1-diphenyl-2-picrylhydrazyl (DPPH) scavenging, hydrogen peroxide (H_2_O_2_), and ferric reducing antioxidant power (FRAP) assays were determined, and the correlations among the results were assessed using Pearson's correlation. The antimicrobial activity was assessed through agar disc diffusion and broth microdilution assays. Phytochemical screening showed that *Z. spinosa* extracts had alkaloids, glycosides, saponins, triterpenoids, phenols, and flavonoids in different abundances. The aerial part-AE extract contained low TPC (30.17 ± 4.24 mg GAE/g) and TFC (7.40 ± 1.02 mg QE/g) and displayed significant antioxidant capacity in the DPPH (IC_50_ = 52.17 ± 7.30 *μ*g/mL), H_2_O_2_ (91.22 ± 2.60 *μ*g/mL), and FRAP (EC_50_ = 98.70 ± 2.21 *μ*g/mL) assays. By contrast, the root-AM extract contained high amounts of TPC (87.72 ± 7.75 mg GAE/g) and TFC (25.60 ± 1.57 mg QE/g). It showed significantly high antioxidant activity with IC_50_ values of 12.33 ± 1.88 *μ*g/mL in the DPPH and 39.37 ± 2.59 *μ*g/mL in the H_2_O_2_ assays, as well as reducing power capacity with an EC_50_ value of 20.82 ± 1.14 *μ*g/mL in the FRAP assay. Both TPC and TFC were exhibited negative correlations (*p* < 0.01) with the IC_50_ and EC_50_ values obtained in the applied antioxidant assays. The aerial part-AM extract showed the highest inhibitory activity against *Staphylococcus aureus* (26.5 ± 0.20 mm), followed by *Shigella flexneri* (19.4 ± 0.40 mm) and *Proteus mirabilis* (17.7 ± 0.49 mm). *S*. *aureus* was the most affected microorganism with a minimum inhibitory concentration of 128 *μ*g/mL against the aerial part-AM extract. Interestingly, all evaluated extracts showed potent antifungal activity against *Candida albicans*. However, aerial part-AM was the most effective, with an inhibition zone of 12.6 ± 0.17 mm. The results concluded that *Z. spinosa* possesses different phytochemicals displaying significant antioxidant and antimicrobial activities, thus lending credence to its use in traditional medicine.

## 1. Introduction

Human beings are either entirely or partially dependent on plants and plant products, both directly and indirectly. For example, medicinal plants are utilized in traditional medicine practices worldwide. Many people in the Kingdom of Saudi Arabia still believe that traditional medicines offer an alternative to modern practices in the realm of disease treatment [1]. The floras of the Kingdom of Saudi Arabia are considered the most affluent areas of biodiversity in the Arabian Peninsula, with an estimated 2250 species belonging to 132 families and 837 genera [2, 3]. Of these, 26% have known medicinal value, with 13% currently being used in traditional medicines and 61% yet to have their therapeutic properties investigated [4]. Interestingly, one-third of Saudi floras, nearly 1000 species, are found in the Asir region. Many plant species common to the Asir region are known to contain medically active constituents and are currently used by the region's inhabitants for disease treatment.

*Zilla spinosa* (L.) Prantl., locally named shibrim or silla, is a perennial, spiny shrub belonging to the family Brassicaceae (Cruciferae), which comprises 321 genera and 3660 species distributed worldwide [5]. It is widespread throughout the Asir region and is familiar to local inhabitants due to its prevalence in traditional medicine. The *Z. spinosa* has been widely employed in traditional medicine for treating several illnesses, including gastrointestinal disorders and diabetes [6], urinary tract pains [7], diarrhea, gall bladder, kidney stones, liver and pancreas pain, respiratory ailments [8], and rheumatism [9, 10]. The plant is also found to have antimicrobial activity [8, 11] and anti-inflammatory, analgesic, and anticancer properties [12]. A previous study [13] has investigated the hepatoprotective effect of *Z. spinosa* extract against carbon tetrachloride-induced acute hepatotoxicity in rats and concluded that the hepatoprotective effect of the extract is attributed to the combined action of flavonoids. Similarly, El-Sharabasy and Mohamed [12] investigated the chemical constituents and biological activity of chloroform extract of *Z. spinosa* aerial parts. They isolated and identified different compounds including three coumarin compounds, bergapten, psoralene, and umbleferone, and two triterpenes, *β*-amyrin and friedelene, in addition to four phytosterols, campesterol, spinasterol, *β*-sitosterol, and stigmasterol, and examined their anti-inflammatory, analgesic, and antimicrobial activities. The obtained results showed significant anti-inflammatory and antimicrobial effects of *Z. spinosa* extract. Moreover, previous phytochemical studies of *Z. spinosa* revealed various classes of phytochemicals including flavonoids, alkaloids, tannins, anthraquinones, saponins, sterols, and triterpenes [8, 11, 14]. Furthermore, Sekkoum et al. [6] analyzed the essential oil obtained from the aerial parts of *Zilla macroptera*, a species collected from Algeria. They identified 72 compounds, with carvone oxide as the main compound.

Despite the wide therapeutic potential presented by *Z. spinosa*, studies on the phytochemical profile, antioxidant, and antimicrobial activities of this plant are still lacking. Consequently, the present study focused on determining the content of total phenolic and flavonoids, evaluating antioxidant and antimicrobial activities of aqueous ethanol and aqueous methanol extracts of the aerial parts and roots of Saudi *Z. spinosa* collected from the Asir region. The present work also aims to provide an overview of this species' chemical and biological properties to develop sufficient background knowledge for continued research into the different extracts of this plant.

## 2. Materials and Methods

### 2.1. Chemicals and Reagents

All chemicals and solvents were of analytical grade and were used without further purification. Folin–Ciocalteu reagent, DPPH, *p*-iodonitrotetrazolium chloride (INT), TPTZ (2,4,6-tripyridyl-s-triazine), gallic acid, quercetin, and the ascorbic acid and antibiotic standards were purchased from Sigma-Aldrich (St. Louis, Missouri, USA). Aluminum chloride (anhydrous powder), hydrogen peroxide (30%), nitric acid, hydrochloric acid, sodium carbonate (anhydrous powder), ferrous sulfate (FeSO_4_.7H_2_O), and ferric chloride were obtained from Merck Co. The internal standards for elemental analysis were prepared from their stock solutions (1000 *μ*g/mL, ULTRA Scientific, North Kingstown, RI, USA).

### 2.2. Plant Materials and Extraction

The aerial parts and root materials of *Z. spinosa* were collected in April 2018 from three locations around Abha City in the Asir region and were then authenticated using the available relevant scientific records of Saudi Arabia Flora [15] and The Plant List [16]. The voucher specimens were deposited at the College of Science, King Khalid University (Abha, Saudi Arabia) for future reference. The plant is not endangered, so no permission was needed for its collection. The plant materials were washed thoroughly to remove soil particles using tap water, followed by distilled water. They were cut into small pieces and air-dried in shade for two weeks at 25–30°C. The dried sample was then powdered and weighed to obtain 220 g of bark and 650 g of the aerial parts of *Z. spinosa*. Powdered sample (100 g) was macerated with 1 L of aqueous ethanol (80%) and aqueous methanol (80%) separately at 25–30°C for three days with random shaking. The macerates were filtered and then evaporated to dryness at 40°C using an IKA R10C S99 Rotary Evaporator. The dried crude extracts were weighed and stored at 4°C until analysis. The extract yield was calculated as a percentage.

### 2.3. Phytochemical Screening

The crude aqueous ethanol extract of each plant sample was subjected to a qualitative phytochemical screening using the methods described by Gul et al. [17]. The results are given as the relative abundance of the respective compound.

### 2.4. Estimation of the Total Phenolic Content

The Folin–Ciocalteu method was applied as previously described [18] to determine the TPC in each plant extract. Then, 2.5 mL of 0.2 N Folin–Ciocalteu reagent was added to 0.5 mL of the plant extracts (0.1 mg/mL in methanol), mixed for 5 min, and then 2.0 mL of a 7.5% aqueous sodium carbonate solution was added. The reactants were then incubated at 30°C for 90 min to allow for color development. The absorbance was measured at 760 nm on a JASCO V-530 UV/Vis spectrophotometer (Tokyo, Japan). This procedure was repeated for the methanolic solutions of gallic acid standards (0, 0.01, 0.02, 0.04, 0.06, 0.08, 0.1, and 0.2 mg/mL) in order to prepare a calibration curve. The TPC was calculated using the regression equation deriving from the standard curve. The results are expressed as mg GAE/g dry weight of the plant extracts.

### 2.5. Estimation of the Total Flavonoid Content

TFC was estimated using an aluminum chloride assay, described by Ordoñez et al. [19] with slight modifications. Briefly, 2 mL of the plant extracts (0.1 mg/mL in methanol) in a test tube was mixed with 2 mL of a 2% methanolic aluminum chloride solution. The test tube was incubated at 30°C for 60 min to allow for color development. Upon completion, the absorbance at 415 nm was measured. A range of quercetin standards (0.01–0.1 mg/mL in methanol) were prepared to produce the calibration curve, which was analyzed in the same manner as the plant extracts. The TFC was calculated using the regression equation derived from the quercetin standard curve. The results are expressed as mg QE/g dry weight of the plant extracts.

### 2.6. Antioxidant Activity

#### 2.6.1. DPPH Radical Scavenging Activity Assay

The DPPH free radical scavenging activity of the plant extracts was determined by following a previously described method [20], with minor modifications. Briefly, 0.5 mL of the plant extracts of various concentrations (50, 100, 200, 300, and 400 *μ*g/mL in methanol) and the ascorbic acid standard were mixed with 2.5 mL of a 0.1 mM methanolic solution of DPPH. The reactants were incubated for 30 min at 30°C. The absorbance was measured at 517 nm using methanol as a blank. The antioxidant activity of the extracts, expressed as inhibition percentage of DPPH free radicals, was calculated using

(1) $$\begin{array}{c} \text{scavenging effect}\left(\%\right)=\frac{\text{absorbance of control }-\text{absorbance of sample}}{\text{absorbance of control}}\times 100. \end{array}$$

The scavenging effect (%) vs. the extract concentration (*μ*g/mL) was plotted as a graph, and the obtained regression equation was used to calculate the IC_50_ value (the concentration of extract or standard that can inhibit 50% of the DPPH).

#### 2.6.2. Hydrogen Peroxide Scavenging Assay

The H_2_O_2_ scavenging activity was determined by the method of Ruch et al. [21] with minor modifications. A 40 mM solution of hydrogen peroxide (30%) was prepared in 0.1 M phosphate buffer (pH 7.4). Then, 2 mL of the plant extract (50, 100, 200, 300, and 400 *μ*g/mL in distilled water) was added to 2 mL of the hydrogen peroxide (40 mM) solution. After 10 min of incubation at 30°C, the absorbance of the reaction mixture was measured at 230 nm against phosphate buffer as a blank. Ascorbic acid in distilled water (50, 100, 200, 300, and 400 *μ*g/mL) was used as a standard. The percentage of H_2_O_2_ scavenging by both the extracts and the standard was calculated using equation (1). The IC_50_ value (the concentration of extract or standard that can inhibit 50% of the H_2_O_2_) was determined from the regression analysis of a plot of scavenging effect (%) against the extract concentration (*μ*g/mL).

#### 2.6.3. Ferric Reducing Antioxidant Power Assay

The antioxidant capacity of each sample was determined using the FRAP assay, as previously described by Benzie and Strain [22] with some modifications. The assay is based on the reduction of the colorless complex of ferric tripyridyltriazine (Fe^3+^-TPTZ) to the blue color complex of ferrous tripyridyltriazine (Fe^2+^-TPTZ) at low pH by the action of the extracts (antioxidants). Briefly, the fresh working FRAP reagent was prepared by mixing 300 mM acetate buffer (pH 3.6) with a solution of 10 mM of TPTZ in 10 mL of a 40 mM HCl and 20 mM FeCl_3_ solution at 10 : 1 : 1 (v/v/v). Various concentrations (50, 100, 200, 300, and 400 *μ*g/mL) of each plant extract or ascorbic acid were prepared; 1 mL of each was added to 2 mL of the reagent and mixed thoroughly. Then, it was incubated at 37°C for 30 min, and the absorbance at 593 nm was determined using acetate buffer as the blank. A standard curve was plotted using various concentrations of FeSO_4_.7H_2_O. The antioxidant capacity of each extract and the standard, i.e., ascorbic acid, was measured and expressed as *μ*M of the Fe^2+^ equivalent, and the concentration of extract or standard that can inhibit 50% of the FRAP capacity (EC_50_) in *μ*g/mL of each plant extract was determined.

### 2.7. Antimicrobial Activity

The plant extracts were tested for their ability to antagonize the growth of select human pathogens. The pathogenic microbes (procured from the Department of Biology, Faculty of Science, KKU) were common Gram-positive (i.e., *Staphylococcus aureus*) and Gram-negative (i.e., *Escherichia coli*, *Shigella flexneri*, *Proteus mirabilis*, and *Klebsiella pneumoniae*) bacteria, as well as the fungus *Candida albicans*.

#### 2.7.1. Agar Disc Diffusion Method

The pathogens were inoculated on a nutrient agar plate and incubated at 30°C for 48 h. The disc diffusion method, as described by Balouiri et al. [23] with slight modifications, was adapted to test the effectiveness of the extract on the selected microorganisms. Paper discs (6.0 mm) were saturated with a crude extract solution (100 *μ*g/mL), dried, and then placed onto prepared test plates. Triplicate plates for each treatment were prepared and incubated for 18–24 h at 25°C and 37°C for the fungi and bacterial strains, respectively. The subsequent zones of inhibition were measured, and the mean diameter in millimeters was calculated. Ampicillin (100 *μ*g/mL) and nystatin (100 *μ*g/mL) were used as positive controls for antibacterial activity and antifungal activity, respectively, and a medium without inoculation was used as a negative control.

#### 2.7.2. Broth Microdilution Method

The MIC for plant extract was determined using the nutrient broth microdilution method as previously described [24], with minor modifications. The method was performed using different concentrations of the plant extract dissolved in dimethyl sulfoxide. An aliquot of 5 *μ*L of the extract solution was added to 95 *μ*L of fresh media followed by the addition of 100 *μ*L of inoculum (2 × 10^6^ CFU/mL) prepared in Mueller-Hinton agar into different wells of a 96-well microplate. Chloramphenicol was used as a reference antibiotic. The microplates were incubated for 24 h at 37°C in a shaking incubator. Then, 10 *μ*L of a 0.5% INT solution was added to each well and incubated for a further 30 min at 37°C. The MIC value, defined as the lowest sample concentration that inhibited complete bacteria growth, was determined for each sample.

### 2.8. Statistical Analysis

All measurements were performed in triplicate, and the results are presented as means ± standard deviations (SDs). GraphPad Prism version 8.0 and IBM SPSS Statistics for Windows, version 21 (IBM Corp., Armonk, N.Y., USA), were used to perform the statistical analyses. One-way analysis of variance (ANOVA) and *t*-tests were employed for comparative studies among the results. A difference was considered statistically significant if *p* < 0.05 or *p* < 0.01. The correlations among the TPC, TFC, and antioxidant activities and between the assays were assessed using Pearson's correlation.

## 3. Results

### 3.1. Extraction Yields

**Figure 1:**
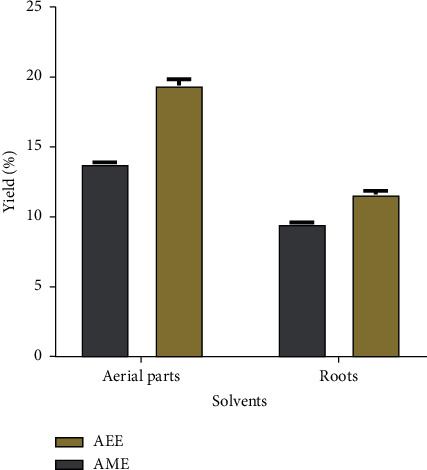
The yield percentage of the aqueous ethanol (AEE) and aqueous methanol (AME) extracts of the aerial and root parts of *Z. spinosa* using the maceration method.

The extraction yield of the aerial parts and root samples of *Z. spinosa* is presented in Figure 1. The mean percentages of the yielded extracts are significantly (*p* < 0.01) different among the plant parts and extractants used and vary from 9.5 ± 0.10% to 19.4 ± 0.44%, with a descending order of aerial parts, aqueous ethanol extract (Ap-AEE) > aerial parts, aqueous methanol extract (Ap-AME) > roots, aqueous ethanol extract (R-AEE) > roots, and aqueous methanol extract (R-AME). The highest percentage of yield was found in Ap-AEE (19.4 ± 0.44% w/w) while the lowest percentage of yield was found in R-AME (9.5 ± 0.10% w/w).

### 3.2. Phytochemical Profiling

Qualitative screening of the phytochemical constituents of the aqueous ethanol extracts from the aerial and root parts of *Z. spinosa* revealed most of the screened phytochemicals in different abundances. The results presented in Table 1 show that the root extract contained significant amounts of alkaloids, phenols, and flavonoids, whereas the extracts of the aerial parts exhibited glycosides and triterpenoids in abundance. The presence of anthraquinones was not detected in either the aerial parts or the root extracts.

### 3.3. Total Phenolic and Flavonoid Contents

**Figure 2:**
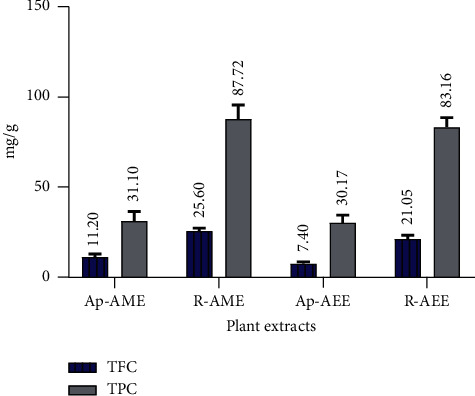
A comparison of the total phenolic content (TPC) and total flavonoid content (TFC) in different extracts of the aerial and root parts of *Z. spinosa*. Ap-AME, aerial parts aqueous methanol extract; R-AME, root aqueous methanol extract; Ap-AEE, aerial parts aqueous ethanol extract; R-AEE, aerial parts aqueous ethanol extract.

In this study, the total phenolic content (TPC) and total flavonoid content (TFC) of the aqueous ethanol (AEE) and aqueous methanol (AME) extracts of the aerial parts and roots of *Z. spinosa* were determined using the Folin–Ciocalteu and aluminum chloride spectrophotometric methods, respectively. Their values were calculated using the linear equations obtained from the gallic acid calibration curve (*y* = 9.6851*x* + 0.0353, *R*^2^ = 0.99) for TPC and from the quercetin calibration curve (*y* = 31.904*x* + 0.0272, *R*^2^ = 0.99) for TFC. A comparison of the amounts of TPC and TFC in the different extracts of the aerial parts and roots of *Z. spinosa* is presented in Figure 2. The amounts of TPC obtained in the aerial parts and root extracts can be ranked in descending order by their mean values: R-AME > R-AEE > Ap-AME > Ap-AEE. A similar trend was also observed for TFC. The TPC and TFC values were significantly (*p* < 0.05) different among plant parts and between the extracts (solvents). Both extracts (AEE and AME) of the roots showed significantly (*p* < 0.05) higher amounts of TPC and TFC compared to the aerial parts. Moreover, the AME of the roots contained the highest TPC (87.72 ± 7.75 mg GAE/g) and TFC (25.60 ± 1.57 mg QE/g) compared to those of AEE (83.16 ± 5.34 mg GAE/g and 21.05 ± 2.18 mg QE/g).

### 3.4. Antioxidant Activity

**Figure 3:**
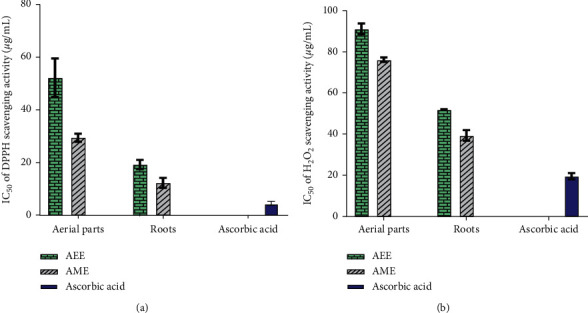
Antioxidant activities of the aerial parts and root extracts of *Z. spinosa* in comparison to the standard, i.e., ascorbic acid: (a) IC_50_ (*μ*g/mL) values of the 1,1-diphenyl-2-picrylhydrazyl (DPPH) assay; (b) IC_50_ (*μ*g/mL) values of the hydrogen peroxide (H_2_O_2_) scavenging assay. AEE, aqueous ethanol extract; AME, aqueous methanol extract.

**Figure 4:**
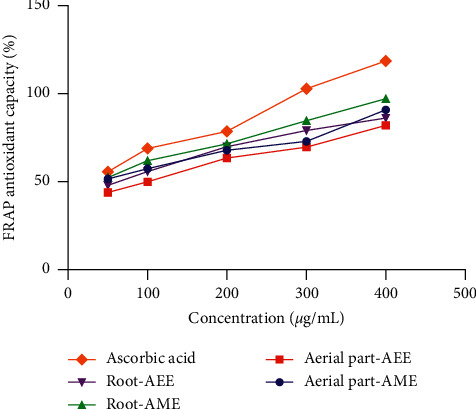
Ferric reducing antioxidant power (FRAP) of the aerial parts and root extracts of *Z. spinosa*.

The AEE and AME extracts from *Z. spinosa* aerial parts and roots were screened for antioxidant activity at five different concentrations using 1,1-diphenyl-2-picrylhydrazyl (DPPH) and hydrogen peroxide (H_2_O_2_) assays to determine free radical scavenging activity and ferric reducing antioxidant power (FRAP) to determine reducing power capacity. The standard antioxidant, i.e., ascorbic acid, was used as the positive control. The IC_50_ (the concentration of extract or standard that can inhibit 50% of the DPPH or H_2_O_2_ capacity) or EC_50_ (the concentration of extract or standard that can inhibit 50% of the FRAP capacity) values were determined from regression analysis of a plot of percent inhibition against the extract concentration (*μ*g/mL). The results indicate a concentration-dependent increase in antioxidant scavenging activity. A comparison of the IC_50_ values (*μ*g/mL) obtained from the DPPH and H_2_O_2_ assays for the different extracts and ascorbic acid is presented in Figure 3. The statistical analysis shows significant differences (*p* < 0.05) in the IC_50_ values between AEE and AME and between the two different plant parts. It may be worth mentioning that in Figure 4, a lower IC_50_ value of the extract means higher free radical scavenging activity. The highest DPPH and H_2_O_2_ scavenging potency was recorded for the AME from the roots (IC_50_ = 12.33 ± 1.88 *μ*g/mL and 39.37 ± 2.59 *μ*g/mL, respectively), and the lowest activity was recorded for the AEE from the aerial parts (IC_50_ = 52.17 ± 7.30 *μ*g/mL and 91.22 ± 2.60 *μ*g/mL).

The reducing power of ferrous ions in the tested extracts is presented in Figure 4. Similar to the radical scavenging capacity, all extracts showed an increasing trend in antioxidant capacity with increasing concentrations. A significant difference in the EC_50_ value between the different extracts and ascorbic acid was also observed. The FRAP capacities of the four extracts were in the order of R-AME > Ap-AME > R-AEE > Ap-AEE. The R-AME extract showed good reducing power capacity with an EC_50_ value of 20.82 ± 1.14 *μ*g/mL, which is comparable to that of ascorbic acid (13.27 ± 0.89 *μ*g/mL).

### 3.5. Antimicrobial Activity

**Figure 5:**
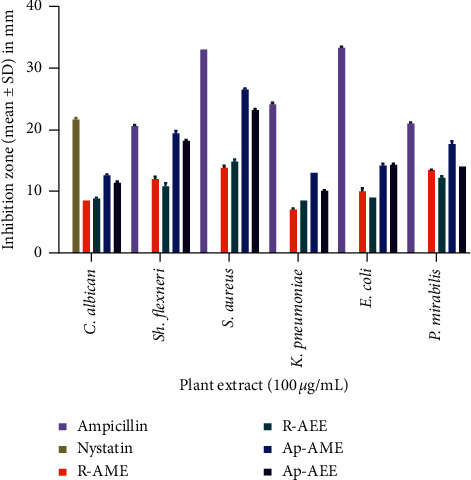
Antimicrobial activity screening of the different *Z. spinosa* extracts.

An agar well diffusion assay was performed using 100 *μ*g/mL of each *Z. spinosa* extract. The results (Figure 5) show that compared with the standard antimicrobials (ampicillin and nystatin), the four extracts (i.e., R-AME, Ap-AME, R-AEE, and Ap-AEE) exhibit appreciable activity against the tested human pathogens. Interestingly, Ap-AME exhibited broad-spectrum activity (100%) against all of the tested pathogens, with inhibition zones ranging from 12.6 ± 0.17 to 26.5 ± 0.20 mm, followed by Ap-AEE, with inhibition zones ranging from 10.1 ± 0.10 to 23.2 ± 0.17 mm; meanwhile, both root extracts exhibited low activity, with inhibition zones ranging from 7.1 ± 0.15 to 14.8 ± 0.35 mm.

Based on the broth microdilution assays, the Gram-positive bacterium (i.e., *S*. *aureus*) was the most susceptible microorganism, with a minimum inhibitory concentration (MIC) of 128 *μ*g/mL against the aqueous methanol extract of the aerial parts of *Z. spinosa*. The Ap-AME extract also showed appreciable activity with MICs of 512 and 1024 *μ*g/mL against the growth of *P. mirabilis* and *K. pneumoniae*, respectively.

### 3.6. Correlation Analysis

**Figure 6:**
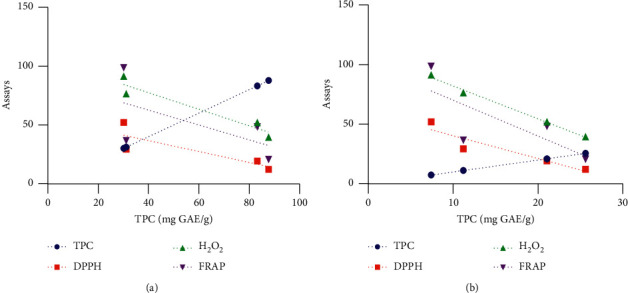
Correlation between TPC and TFC and the antioxidant capacities of the *Z. spinosa* aerial parts and root extracts: (a) linear correlation between TPC and the antioxidant assays; (b) linear correlation between TFC and the antioxidant assays.

Pearson's correlation analysis was performed to assess the correlations between TPC and TFC and the IC_50_ or EC_50_ values of the antioxidant assays as well as between the assays.  Table 2 shows the correlation coefficients (*R*) obtained between TPC and TFC and the antioxidant capacities, and their linear correlations are presented in Figure 6. A strong significantly positive (*R* = 0.947, *p* < 0.01) correlation between TPC and TFC was observed. Significant positive correlations were found between the antioxidant assays. The lowest correlation was found between the FRAP and H_2_O_2_ assays (*R* = 0.800, *p* < 0.01). Pearson's correlation coefficient between the TPC and the antioxidant capacities revealed that TPC exhibited high negative correlations with the IC_50_ values obtained in the H_2_O_2_ (*R* = −0.940, *p* < 0.01) and DPPH (*R* = −0.817, *p* < 0.01) assays and a medium correlation with the EC_50_ value obtained in the FRAP assay (*R* = −0.586, *p* < 0.05). Similarly, the TFC exhibited a negative and high correlation with the IC_50_ values obtained in the H_2_O_2_ (*R* = −0.969, *p* < 0.01) and DPPH (*R* = −0.881, *p* < 0.01) assays, though it also had a high negative correlation (*R* = −0.747, *p* < 0.01) with the EC_50_ value obtained from the FRAP assay.

## 4. Discussion

### 4.1. Extraction Yield

The extraction yield, defined as the mass of extract recovered compared to the initial amount of plant material, is a measure of the solvent's efficiency to extract the chemical constituents of the plant materials. The mean values of the yielded extracts, presented as percentages (%), were significantly (*p* < 0.05) different among the plant parts and solvents used for extraction. The extraction yields (Figure 1) show that the highest percentage of yield was obtained in the aqueous ethanol extracts of both the aerial parts and the roots (19.4% w/w and 11.6% w/w, respectively). The results indicate that the aqueous ethanol extracted higher yield than the aqueous methanol. Due to the fact that the extraction yield increased with the increasing polarity of the solvent used in the extraction and that water enhances the polarity of the organic solvents, a higher yield was expected using the aqueous methanol rather than the aqueous ethanol. The present results indicate that the higher extraction yield obtained using the aqueous ethanol may be attributed to the presence of less polar constituents that were more readily extracted using aqueous ethanol than aqueous methanol.

### 4.2. Phytochemical Profiling

The phytochemical screening results presented in Table 1 show the presence of alkaloids, glycosides, saponins, triterpenoids, tannins, phenols, and flavonoids in different abundances in the extracts of *Z. spinosa* aerial parts and roots. The presence of anthraquinones was not detected in either extract. The phytochemical profile of *Z. spinosa* obtained in this study resembled that from previous reports [8, 11, 14]. The presence of phytochemical constituents such as flavonoids, glycosides, saponins, alkaloids, phenols, tannins, and terpenoids in this plant likely contributes to its biological activity and may provide the basis for its uses in traditional medicine. These phytochemicals have been proven to display a wide range of pharmacological properties such as antibacterial, antifungal, antiviral, anticancer, anti-inflammatory, antitumor, and antithrombotic as well as antioxidant [25]. For example, alkaloids, phenols, tannins, and flavonoids are known to have a wide range of biological activities, including antibacterial, anti-inflammatory, and antioxidant activities [25, 26]. Thus, the presence of such compounds in the aerial parts and root extracts support the traditional use of *Z. spinosa* in the treatment of gastrointestinal disorders. The results of this study show that alkaloids are present in abundance in the root extracts. It is reported that alkaloids derived from plants exhibit antimicrobial and anticancer effects [27]. Moreover, the phytochemical screening results also show the presence of tannins, phenols, and flavonoids in abundance in the root extracts. Phenolic and flavonoid compounds are known for their antioxidant and antidiabetic activities [28]. These findings can explain the use of the whole plant of *Z. spinosa* for diabetes treatment.

### 4.3. Total Phenolic and Total Flavonoid Contents

Polyphenols (i.e., flavonoids and phenolic compounds) are naturally occurring antioxidants that are believed to play a major role in the prevention of various diseases such as diabetes, cardiovascular diseases, neurodegenerative diseases, liver disease, and cancers [29]. Consequently, the determination of TPC and TFC is necessary for predicting a plant extract's antioxidant capacity. Figure 2 shows the presence of significant TPC and TFC in the four extracts of *Z. spinosa*. R-AME possessed the highest TPC (87.72 ± 7.75 mg GAE/g), whereas Ap-AEE had the lowest TPC (30.17 ± 4.24 mg GAE/g). Similarly, the maximum TFC was recorded in R-AME (25.60 ± 1.57 mg QE/g) and the lowest TFC was present in Ap-AEE (7.40 ± 1.02 mg QE/g). These results indicate that the solvent type exerted significant influence on the TPC and TFC extraction profiles. In this study, AME was found to be the best extractant for phenolic compounds compared to AEE. Importantly, the magnitude of the values for TPC and TFC obtained for the *Z. spinosa* aerial parts and root extracts are consistent with those obtained for phenols and flavonoids during the preliminary phytochemical screening (Table 1). These TPC and TFC values are considered to be lower when compared to the findings of Bouchouka et al. [9], who analyzed the fruits extract of *Z. spinosa* collected from the Algerian Sahara. Meanwhile, another study [10] tested the aerial parts of *Z. spinosa* from Algeria, indicating much lower values compared with our results. These variations in TPC and TFC values might be due to the distinct geographical conditions of the areas being studied, considering plants produce polyphenols as a response to environmental factors (e.g., light, temperature, and competition), as well as edaphic factors such as soil type [30]. Moreover, the types and amounts of polyphenols vary depending on the species and geographic origin and, importantly, the types of solvents used for their extractions. The present estimations indicate that *Z. spinosa* contains a significant amount of phenolic and flavonoid content as well as considerable amounts of other essential phytochemicals. The presence of valuable constituents could account for the pharmacological activities reported for *Z. spinosa* and might explain its extensive use in traditional medicine.

### 4.4. Antioxidant Activity

The aerial parts and root extracts of *Z. spinosa* were screened for antioxidant activity using the DPPH scavenging, H_2_O_2_, and FRAP assays. The results show that all *Z. spinosa* extracts had strong antioxidant activities corresponding to their IC_50_ values that ranged from 12.33 ± 1.88 to 52.17 ± 7.30 *μ*g/mL in the DPPH assay and from 39.37 ± 2.59 to 91.22 ± 2.60 *μ*g/mL in the H_2_O_2_ assay, while their EC_50_ values ranged from 20.82 ± 1.14 to 98.70 ± 2.21 *μ*g/mL in the FRAP assay. A lower IC_50_ or EC_50_ value corresponds to a higher antioxidant capacity, and according to a previous report, a plant extract with an IC_50_ or EC_50_ of <100 *μ*g/mL is classified as a strong antioxidant [31]. The outcomes herein indicate that the antioxidant potential displayed by the aqueous methanol extract of the roots was the highest. It displayed scavenging activity with IC_50_ values of 12.33 ± 1.88 *μ*g/mL in the DPPH and 39.37 ± 2.59 *μ*g/mL in the H_2_O_2_ assays as well as reducing power capacity with an EC_50_ value of 20.82 ± 1.14 *μ*g/mL in the FRAP assay, which were comparable to those displayed by the standard antioxidant, i.e., ascorbic acid (Figures 3 and 4). The present findings are in agreement with those previously reported for *Z. spinosa* [9]. These appreciable radical scavenging activities of *Z. spinosa* could be due to the relatively high TPC and TFC in the roots and aerial parts of this plant. Notably, the plant extracts' phenolic/flavonoid contents were mainly correlated with the plant's antioxidant capacities [32, 33]. Thus, the strong antioxidant activity observed in the current investigation is in accordance with the high TPC and TFC of the *Z. spinosa* extracts. Owing to its strong antioxidant activity, the use of *Z. spinosa* for the treatment of gastrointestinal disorders and diabetes has been reported [6].

### 4.5. Antimicrobial Activity

As demonstrated by Ruiz-Ruiz et al. [34], the inhibition zones of 14.0–19.0 mm and >19.0 mm can be classified as active and very active, respectively. Thus, *Z. spinosa* has promising antibacterial activity. Higher inhibitory activity was observed in the Ap-AME against *S. aureus* (26.5 ± 0.20 mm), followed by *Sh. flexneri* (19.4 ± 0.40 mm), *P. mirabilis* (17.7 ± 0.49 mm), *E. coli* (14.2 ± 0.35 mm), and *K. pneumoniae* (13.0 ± 0.00 mm). Ap-AEE also exhibited potent antibacterial activity but with lower inhibition zones when compared to Ap-AME. Meanwhile, both root extracts (R-AEE and R-AME) exhibited activities with inhibition zones ranging from 7.1 ± 0.15 to 14.8 ± 0.35 mm against the tested bacterial strains. These findings are comparable to those that previously revealed antibacterial activities for this plant [8, 9, 11, 12]. Interestingly, all *Z. spinosa* extracts showed potent antifungal activity against *C*. *albicans* (Figure 5). However, Ap-AME was the most effective, with an inhibition zone of 12.6 ± 0.17 mm, followed by Ap-AEE (11.4 ± 0.20 mm), whereas nystatin, the reference antifungal drug, inhibited the growth of *C*. *albicans* with a 21.7 ± 0.29 mm zone of inhibition. These results disagree with those of EL-Sharabasy and Mohamed [12], who reported a negative antifungal effect of the aerial parts of *Z. spinosa* against both *C*. *albicans* and *Aspergillus flavus*.

Based on the antimicrobial assays, the Gram-positive bacterium (i.e., *S*. *aureus*) was the most susceptible microorganisms, with a 26.5 ± 0.20 mm zone of inhibition and an MIC of 128 *μ*g/mL against the Ap-AME extract of *Z. spinosa*. *S. aureus* is an opportunistic pathogen and one of the leading causes of community-acquired infections throughout the world [35]. It is susceptible to the reference antibiotic chloramphenicol, with an MIC of 8 *μ*g/mL. The obtained findings are consistent with those of Alghanem et al. [11], who reported that *S. aureus* is the bacteria most affected by different extracts of *Z. spinosa*. The Ap-AME extract of *Z. spinosa* also showed appreciable activity, with MICs of 512 and 1024 *μ*g/mL against the growth of *P. mirabilis* and *K. pneumoniae*, respectively. The considerable antimicrobial activity of *Z. spinosa* may be attributable to its various constituent phytochemicals (Table 1). Several reports have noted that the antimicrobial activities observed using plant extracts result from phytochemicals such as tannins, flavonoids, alkaloids, glycosides, and terpenoids [36]. Additionally, the antibacterial activity of *Z. spinosa*, particularly its anti-*S. aureus* effect, justifies the use of this plant in traditional medicine for treating respiratory ailments and their symptoms [8].

### 4.6. Correlation Analysis

Regarding the relationships between TPC and TFC and antioxidant activities, the studied parameters were significantly correlated, as shown by Pearson's correlation matrix in Table 2. The highly significant positive (*R* = 0.947, *p* < 0.01) correlation between TPC and TFC observed in the present study was expected because flavonoids can be classified as a group of phenolic compounds. The TPC in the extracts of *Z. spinosa* exhibited a significantly high negative correlation (*p* < 0.01) with the IC_50_ values obtained in the DPPH and H_2_O_2_ assays but exhibited a low negative correlation with the EC_50_ value obtained in the FRAP assay (*R* = –0.586, *p* < 0.05). A similar trend was observed with TFC and the IC_50_ and EC_50_ values obtained in the applied assays. These results indicate that increases in the TPC and TFC decrease the IC_50_ or EC_50_ values, resulting in high antioxidant activity. The present findings are in agreement with previous reports that indicate that TPC and TFC are responsible for high antioxidant activity [37]. Significant positive correlations were also detected between the antioxidant assays, which indicates good agreement between the antioxidant capacities obtained using these assays. The IC_50_ values obtained in the DPPH and H_2_O_2_ assays and the EC_50_ value from the FRAP assay along with the comparison of correlation coefficients suggest that TPC and TFC both contribute similarly to the observed antioxidant activity of the *Z. spinosa* extracts.

## 5. Conclusions

The present study demonstrated that *Z. spinosa* extracts exert antimicrobial activity against various human pathogens and indicate that it might be valuable in treating infectious diseases caused by microorganisms. Furthermore, *Z. spinosa* extracts were found to contain a high amount of total phenolic and total flavonoids. Likewise, the extracts revealed significant antioxidant activities against various tests: DPPH, H_2_O_2,_, and FRAP. In general, the results prove the effectiveness of the plant for its potent antioxidant and antimicrobial activities. Accordingly, the positive values of the plant regarding its application in traditional medicine have been confirmed. The present findings are expected to be equally beneficial to everyone dealing with medicinal plant benefits across the world. However, further studies, including in vitro and in vivo investigations, are needed to confirm their antimicrobial and antioxidant activities, identify and isolate the active compounds, and elucidate their pharmacological properties. Consequently, further studies are being carried out by the authors to isolate the flavonoid compounds of these extracts and to test their effectiveness as anticancer agents.

## Figures and Tables

**Table 1:** Preliminary phytochemical profile of the aerial parts and roots of *Z. spinosa*.

Phytochemical components	Test/reagent	*Z. spinosa*	
		Aerial parts	Roots
Alkaloids	Mayer	−/+	+/+
	Wagner		

Glycosides	Liebermann	+/+	–/+
	Keller–Kiliani		

Saponins	Foam test	+	+

Triterpenes/sterols	Liebermann–Burchard	+/+	−/+
	Salkowski		

Tannins and phenols	Ferric chloride	−/+	+/+
	Lead acetate		

Flavonoids	Alkaline reagent	−/+	+/+
	Shinoda		

Anthraquinones	Borntrager	−	−

+, one test was conducted and it gave a positive result; −, one test was conducted and it gave a negative result; +/+, two different tests were conducted and both gave positive results; −/+, two different tests were conducted and one gave a negative and the other gave a positive result; −/−, two different tests were conducted and both gave negative results.

**Table 2:** Pearson's correlation coefficients of the antioxidant capacities, TPC, and TFC.

Parameters	TPC	TFC	DPPH	H_2_O_2_
TPC	0.947*∗*			
DPPH	−0.817*∗*	−0.881*∗*		
H_2_O_2_	−0.940*∗*	−0.969*∗*	0.943*∗*	
FRAP	−0.586*∗∗*	−0.747*∗*	0.908*∗*	0.800*∗*

*∗*Correlation is significant at *p* < 0.01; *∗∗* correlation is significant at *p* < 0.05.

## Data Availability

The data sets used during the current study are available from the corresponding author upon reasonable request.

## References

[1] Al Akeel M. M., Al Ghamdi W. M., Al Habib S., Koshm M., Al Otaibi F. (2018). Herbal medicines: Saudi population knowledge, attitude, and practice at a glance. *Journal of Family Medicine and Primary Care*.

[2] El-shabasy A. (2016). Survey on medicinal plants in the flora of Jizan region, Saudi Arabia. *International Journal of Botany Studies*.

[3] Yusuf M., Al-Oqail M. M., Al-Sheddr E. S., Al-Rehaily A. J., Rahman M. A. (2014). Diversity of medicinal plants in the flora of Saudi Arabia 3: an inventory of 15 plant families and their conservation management. *International Journal of Environment*.

[4] Sher H., Aldosari A. (2012). Overview on the ecological and geographical appraisal of important medicinal and aromatic plants: an endangered component in the flora of Saudi Arabia. *Scientific Research and Essays*.

[5] Al-Shehbaz I. A. (2012). A generic and tribal synopsis of the Brassicaceae (Cruciferae). *TAXON*.

[6] Sekkoum K., Belboukhari N., Cheriti A., Aboul-Enein H. (2015). Chemical composition of the essential oil of *Zilla macroptera* coss. From Algerian sahara. *Current Bioactive Compounds*.

[7] Sekkoum K., Cheriti A., Taleb S., Bourmita Y., Belboukhari N. (2011). Traditional phytotherapy for urinary diseases in Bechar district (South West of Algeria). *Electronic Journal of Environmental, Agricultural and Food Chemistry*.

[8] Berreghioua A., Cheriti A., Belboukhari N. (2014). Antibacterial activity of *Zilla macroptera* extracts from Algerian Sahara. *PhytoChem & BioSub Journal*.

[9] Bouchouka E., Djilani A., Bekkouche A. (2012). Antibacterial and antioxidant activities of three endemic plants from Algerian sahara. *Acta Scientiarum Polonorum. Technologia Alimentaria*.

[10] Keffous F., Belboukhari N., Djaradi H., Cheriti A., Sekkoum K., Aboul-Enein H. (2016). Total antioxidant capacity, reducing power and cyclic voltammetry of *Zilla macroptera* (Brassicaceae) aqueous extract. *Current Bioactive Compounds*.

[11] Alghanem S. M., Al-hadithy O. N., Milad M. (2018). Phytochemical analysis and antimicrobial activity of *Zilla spinosa* extracts against pathogenic microorganisms. *Journal of Medicinal Botany*.

[12] EL-Sharabasy F., Mohamed N. Z. (2013). Chemical constituents and biological activity from chloroform extract of *Zilla spinosa*. *International Journal of Pharmacy and Pharmaceutical Sciences*.

[13] El-Toumy S. A., El-Sharabasy F. S., Ghanem H. Z., El-Kady M. U., Kassem A. F. (2011). Phytochemical and pharmacological studies on *Zilla spinosa*. *Australian Journal of Basic and Applied Sciences*.

[14] El-sawi S. A., Motawe H. M., Ahmad S. S., Ibrahim M. E. (2018). Survey and assessment of chemical composition and biological activity of some wild plants growing in the Egyptian eastern desert. *Journal of Materials and Environmental Sciences*.

[15] Chaudhary S. A. (2001). *Flora of the Kingdom of Saudi Arabia (Volume II)*.

[16] The Plant List 2013, Version 1.1, 2018, http://www.theplantlist.org/

[17] Gul R., Jan S. U., Faridullah S., Sherani S., Jahan N. (2017). Preliminary phytochemical screening, quantitative analysis of alkaloids, and antioxidant activity of crude plant extracts from *Ephedra intermedia* indigenous to balochistan. *The Scientific World Journal*.

[18] Nabavi S. M., Ebrahimzadeh M. A., Nabavi S. F., Hamidinia A., Bekhradnia A. R. (2008). Determination of antioxidant activity, phenol and flavonoid content of *Parrotia persica mey*. *Pharmacologyonline*.

[19] Ordoñez A., Gomez J., Vattuone M., Lsla M. (2006). Antioxidant activities of *Sechium edule* (Jacq.) swartz extracts. *Food Chemistry*.

[20] Eshwarappa R. S., Iyer R. S., Subbaramaiah S. R., Richard S. A., Dhananjaya B. L. (2014). Antioxidant activity of *Syzygium cumini* leaf gall extracts. *BioImpacts*.

[21] Ruch R. J., Cheng S.-j., Klaunig J. E. (1989). Prevention of cytotoxicity and inhibition of intercellular communication by antioxidant catechins isolated from Chinese green tea. *Carcinogenesis*.

[22] Benzie I. F. F., Strain J. J. (1999). Ferric reducing/antioxidant power assay: direct measure of total antioxidant activity of biological fluids and modified version for simultaneous measurement of total antioxidant power and ascorbic acid concentration. *Oxidants and Antioxidants Part A*.

[23] Balouiri M., Sadiki M., Ibnsouda S. K. (2016). Methods for in vitro evaluating antimicrobial activity: a review. *Journal of Pharmaceutical Analysis*.

[24] Tomida J., Oumi A., Okamoto T. (2013). Comparative evaluation of agar dilution and broth microdilution methods for antibiotic susceptibility testing ofHelicobacter cinaedi. *Microbiology and Immunology*.

[25] Bribi N. (2018). Pharmacological activity of alkaloids: a review. *Asian Journal of Research in Botany*.

[26] Cowan M. M. (1999). Plant products as antimicrobial agents. *Clinical Microbiology Reviews*.

[27] Thawabteh A., Juma S., Bader M. (2019). The biological activity of natural alkaloids against herbivores, cancerous cells and pathogens. *Toxins*.

[28] Tanase C., Coșarcă S., Muntean D.-L. (2019). A critical review of phenolic compounds extracted from the bark of woody vascular plants and their potential biological activity. *Molecules*.

[29] Arora I., Sharma M., Tollefsbol T. O. (2019). Combinatorial epigenetics impact of polyphenols and phytochemicals in cancer prevention and therapy. *International Journal of Molecular Sciences*.

[30] Lopes C., Pereira E., Soković M. (2018). Phenolic composition and bioactivity of *Lavandula pedunculata* (Mill.) Cav. samples from different geographical origin. *Molecules*.

[31] Fidrianny I., Anggraeni N. A. S., Insanu M. (2018). Antioxidant properties of peels extracts from three varieties of banana (Musa sp.) grown in West Java-Indonesia. *International Food Research Journal*.

[32] Kelly E. H., Dennis J. B., Anthony R. T. (2002). Flavonoid antioxidants: chemistry, metabolism and structure-activity relationships. *The Journal of Nutritional Biochemistry*.

[33] Procházková D., Boušová I., Wilhelmová N. (2011). Antioxidant and prooxidant properties of flavonoids. *Fitoterapia*.

[34] Ruiz-Ruiz J. C., Ramón-Sierra J., Arias-Argaez C., Magaña-Ortiz D., Ortiz-Vázquez E. (2017). Antibacterial activity of proteins extracted from the pulp of wild edible fruit of *Bromelia pinguin L.*. *International Journal of Food Properties*.

[35] Fayyaz M., Mirza I. A., Ahmed Z., Abbasi S. A., Hussain A., Ali S. (2013). In vitro susceptibility of chloramphenicol against methicillin-resistant *Staphylococcus aureus*. *Journal of the College of Physicians and Surgeons—Pakistan: JCPSP*.

[36] Mujeeb F., Bajpai P., Pathak N. (2014). Phytochemical evaluation, antimicrobial activity, and determination of bioactive components from leaves of *Aegle marmelos*. *BioMed Research International*.

[37] Zengin G., Mahomoodally M. F., Paksoy M. Y. (2019). Phytochemical characterization and bioactivities of five Apiaceae species: natural sources for novel ingredients. *Industrial Crops and Products*.

